# Combining Augmented Radiotherapy and Immunotherapy through a Nano-Gold and Bacterial Outer-Membrane Vesicle Complex for the Treatment of Glioblastoma

**DOI:** 10.3390/nano11071661

**Published:** 2021-06-24

**Authors:** Mei-Hsiu Chen, Tse-Ying Liu, Yu-Chiao Chen, Ming-Hong Chen

**Affiliations:** 1 Department of Internal Medicine, Far Eastern Memorial Hospital, New Taipei 220, Taiwan; michelle8989@gmail.com; 2 Department of Biomedical Engineering, Ming Chuang University, Taoyuan 333, Taiwan; 3 Department of Biomedical Engineering, National Yang Ming Chiao Tung University, Taipei 112, Taiwan; tyliu5@ym.edu.tw (T.-Y.L.); ian53011@gmail.com (Y.-C.C.); 4 Graduate Institute of Nanomedical and Medical Engineering, Taipei Medical University, Taipei 110, Taiwan; 5 Department of Neurosurgery, Wang Fang Hospital, Taipei Medical University, Taipei 116, Taiwan

**Keywords:** glioblastoma, gold nanoparticles, outer membrane vesicles, immunotherapy, radioenhancer

## Abstract

Glioblastoma, formerly known as glioblastoma multiforme (GBM), is refractory to existing adjuvant chemotherapy and radiotherapy. We successfully synthesized a complex, Au–OMV, with two specific nanoparticles: gold nanoparticles (AuNPs) and outer-membrane vesicles (OMVs) from *E. coli*. Au–OMV, when combined with radiotherapy, produced radiosensitizing and immuno-modulatory effects that successfully suppressed tumor growth in both subcutaneous G261 tumor-bearing and in situ (brain) tumor-bearing C57BL/6 mice. Longer survival was also noted with in situ tumor-bearing mice treated with Au–OMV and radiotherapy. The mechanisms for the successful treatment were evaluated. Intracellular reactive oxygen species (ROS) greatly increased in response to Au–OMV in combination with radiotherapy in G261 glioma cells. Furthermore, with a co-culture of G261 glioma cells and RAW 264.7 macrophages, we found that GL261 cell viability was related to chemotaxis of macrophages and TNF-α production.

## 1. Introduction

Glioblastoma (Grade IV), previously known as glioblastoma multiforme (GBM), is the most malignant brain tumor. Despite the great technological advances in imaging, surgery, and adjuvant therapies, the median survival is 14–16 months after diagnosis, and the 5-year overall survival is only 9.8% [1]. In addition, its infiltrative nature is one of the main reasons for tumor recurrence. Current glioblastoma therapy represents a combination of surgery, radiation, and chemotherapy. There are two FDA-approved glioblastoma drugs: one is temozolomide, a DNA alkylating agent, and the other is bevacizumab, a humanized monoclonal antibody IgG1. Both of them are not effective enough; therefore, there is a constant search for new treatments with improved efficiency and less adverse effects [2,3]. For non-surgical treatment of glioblastoma, enhancement of radiotherapy and better targeting of pharmaceutical agents to tumors through immunotherapy were attempted to improve survival. Metal nanoparticles have been widely used in clinical practice, including diagnostic, therapeutic (radiation dose enhancers, hyperthermia inducers, drug delivery vehicles, vaccine adjuvants, photosensitizers, and enhancers of immunotherapy), and theranostic (combining both diagnostic and therapeutic) applications [4]. Gold nanoparticles (AuNPs) are one of the promising agents. They have several advantages: biocompatibility, well-established methods for synthesis in a wide range of sizes, and the possibility of surface coating with many different molecules to provide, for example, surface charge or interactivity with serum proteins [5]. Safety is the most important concern for all clinical applications. Radiotherapy cannot spare 100% of the healthy tissues neighboring cancerous ones. In order to overcome these limitations, combinations of therapeutic modalities have been proposed, and have already been showing promising results with AuNPs [6,7]. In addition, AuNPs are able to target the tumor by passive targeting (enhanced permeability and retention (EPR) effect and mononuclear phagocyte system (MPS) escape) and active targeting (tumor cell targeting and stimuli-response) [8]. Furthermore, photoexcitation of the AuNPs would lead to the generation of reactive oxygen species (ROS), which are known to play a central role in the photodymamic therapy of cancer [9]. Bacterial outer-membrane vesicles (OMVs) are naturally produced from all Gram-negative bacteria and have nano-sized, lipid-bilayered vesicular structures composed of various immunostimulatory components. An OMV-based vaccine is being clinically used as a meningococcal group B vaccine under the trade name Bexsero. Furthermore, studies have revealed the remarkable ability of OMVs to effectively induce long-term antitumor immune responses without notable adverse effects [10]. In this study, we mixed AuNPs and OMVs into a co-suspension of Au–OMV and tested its safety and effects on glioblastoma through the combination of augmented radiotherapy and immunotherapy.

## 2. Materials and Methods

### 2.1. Preparation and Characterization of Au–OMV

*E. coli* (MAX Efficiency^TM^ DH5α Competent Cells, Thermo Fisher, Waltham, MA, USA), were cultured for 16 h on lysogeny broth (1% tryptone, 0.5% yeast extract, 1% NaCl, pH 7.0) at 37 ${}^{\circ }C$ with shaking (180 rpm) until the OD600 reached 0.8–1. The cultured cells were pelleted twice at 4000×*g* for 10 min. The supernatant was filtered with a filter having 0.45 μm pore size and was concentrated using a 100 kDa hollow fiber membrane (Amicon, Millipore, Kenilworth, NJ, USA). The concentrate was filtered again using a 0.22 μm filter and was pelleted by ultracentrifugation at 150,000×*g* in a SW 41 Ti rotor (Beckman Coulter Inc., Brea, CA, USA) for 3 h. The pellet was suspended in 50% iodixanol, and we used buoyant density gradients of 10%, 40%, and 50% iodixanol layers at 200,000×*g* for 2 h. The fractions containing bacterial OMVs and extracellular vesicles were collected from the third fraction from the top layer. The purified OMVs were filtered with 0.22 m filters to avoid any bacteria or cell debris contamination. Protein concentration was determined using Bradford assay. The sample was aliquoted and stored at −80 ${}^{\circ }C$ until use [11]. For AuNPs synthesis, we dissolved 1 mg HAuCl_4_ in 90 mL deionized water and heated the mixture to boil. The reducing solution, 500 μL 250 mM sodium citrate, was added until the light-yellow solution turned to red. We kept stirring for another 30 min and measured the particle size. The solution containing particles was stored at 4 ${}^{\circ }C$. For the preparation of Au–OMV, solutions containing Au and OMV particles were mixed (at concentrations of 200 μg mL^−1^ and 2 μg mL^−1^ for Au and OMV, respectively) thoroughly in a homogenizer to form a stable co-suspension without forming aggregations of particles.

The morphologies of Au nanoparticles, OMV, and Au–OMV were examined using surface (JEOL, JSM-7600F, Tokyo, Japan) and transmission electron microscopy (JEOL, JEM-2000EX II, Tokyo, Japan). Size distribution was measured using dynamic light scattering at a scattering angle of $90^{\circ }$ using a Zetasizer nano ZS90 (Malvern Instruments, Worcestershire, UK) at 25 ${}^{\circ }C$.

### 2.2. Cell Culture

GL261 mouse glioma cells, C8D1A mouse astrocytes, B.end3 mouse endothelial cells, and RAW264.7 mouse macrophages were grown in 90% Dulbecco’s modified eagle medium (DMEM) supplemented with 10% fetal bovine serum (FBS) and 4 mM glutamine in 75T culture plates at 37 ${}^{\circ }C$ in a 5% CO_2_-containing atmosphere.

### 2.3. Cell Viability Test

Cells were seeded in 98-well plates and incubated for 24 h. After washing three times with phosphate-buffered saline (PBS), OMV and AuNPs at various concentrations (diluted with FBS-containing culture medium) were added to the plates and incubated for 24 h. After washing with PBS, 20X diluted PrestoBlue^®^ reagent was added and reacted with cells for 20 min. Viable cells were evaluated using TECAN Sunrise ELISA Reader (TECAN, Zurich, Switzerland) at excitation/emission (Ex/Em) 560/590 nm.

### 2.4. The Combination of Au–OMV and Radiotherapy Applied to the Co-Culture of GL261 Glioma Cells and RAW264.7 Macrophages

#### 2.4.1. Cell Viability

In total, 3 × 10^5^ GL261 mouse glioma cells were seeded in 6 wells until well attached. After washed with PBS, cells were incubated with 5 μg mL^−1^ OMV and 400 μg mL^−1^ AuNP for 5 h. X-ray radiation (6 MeV, a dose rate of 100 rad/min; and the total dose of X-ray radiation was 2 Gy in a single fraction) was given to the cells, and the 0.4 μm pored upper chambers of transwells with 1 × 10^6^ RAW264.7 mouse macrophages were inserted and co-cultured for 24 h. PrestoBlue^®^ Cell Viability Reagent was used to evaluate the viability of the cells using TECAN Sunrise ELISA Reader at Ex/Em 560/590 nm.

#### 2.4.2. Macrophage Migration (Chemotaxis Assay)

GL261 mouse glioma cells were seeded in 12 wells with a density of 5 × 10^5^/well. After well attached, cells were incubated with 2 μg mL^−1^ OMV and 400 μg mL^−1^ AuNP for 5 h. X-ray radiation (6 MeV, a dose rate of 100 rad/min) was given to the cells, and the total dose of X-ray radiation was 2 Gy in a single fraction. On the other hand, 1 × 10^6^ RAW264.7 mouse macrophages in 1 mL PBS were labeled with 5 μL Vybrant^TM^ DiD (red for macrophages) Cell Labeling Solution at 37 ${}^{\circ }C$ for 20 min. After being centrifuged at 15,000 rpm for 5 min, RAW264.7 cell lysates were resuspended in PBS and were placed in 3 μm pored upper chambers of transwells (inserts) with a density of 5 × 10^5^/well at 37 ${}^{\circ }C$ and co-cultured for 24 h. After washing with PBS for 3 times, cells were fixed with 4% formalin for 10 min and then 0.1% Triton X-100 was added. After washing with PBS for 3 times, Alexa Fluor^TM^ 488 phalloidin (Ex/Em 493/519, green for actin) was added and the solutions were incubated for 30 min. Furthermore, H33342/DAPI (Ex/Em 358/519, blue for nucleus) was added, and then all were observed with the ZEISS LSM 880 (Zeiss, Oberkochen, Germany) confocal microscope.

### 2.5. Intracellular ROS Detection Using Flow Cytometry

In total, 3 × 10^5^ GL261 mouse glioma cells were seeded in 6-well plates until well attached. After washed with PBS, cells were incubated with 5 μg mL^−1^ OMV and 400 μg mL^−1^ AuNP for 5 h. After washed with PBS, Trypsin was added for the detachment of cells. The cell lysate was centrifuged with 1000 rpm for 5 min. After discarding the supernatant, 100 μL CellROX^®^ Deep Red Reagent was added to the centrifuged cells. X-ray (6 MeV, a dose rate of 100 rad/min) was given to the cells and the total dose of X-ray radiation was 2 Gy in a single fraction. After washed with 900 μL PBS, cells were dissolved in 1 μL medium and subjected to flow cytometry (Beckman Coulter CytoFLEX, Beckman Coulter Inc., Brea, CA, USA) for the detection of ROS fluorescence at Ex/Em 495/529 nm.

### 2.6. Confocal Microscopic ROS Detection

GL261 mouse glioma cells were seeded in a Ultra-Low Attachment Surface 6-well plate with a density of 1 × 10^6^ cells/well and cultured with Au–OMV complex overnight. X-ray radiation (6 MeV, a dose rate of 100 rad/min) was given to the cells, and the total dose of X-ray radiation was 2 Gy in a single fraction. The live cells were incubated with 100 μL of CellROX^TM^ Deep Red Reagent (5 μM; Ex/Em 640/665, pink for ROS), 100 μL of H33342/DAPI (Ex/Em 358/519, blue for nucleus), and Alexa Fluor^TM^ 488 phalloidin (Ex/Em 493/519, green for actin) for 1 h and observed under a Zeiss LSM 880 confocal microscope.

### 2.7. Western Blot

Cells were washed three times using PBS at 24 h after treatment and subsequently added to lysis buffer for 5 min. The protein concentration in each cell lysate was then quantified and adjusted depending on the concentration. The proteins were further separated using sodium dodecyl sulfate polyacrylamide gel electrophoresis (SDS-PAGE) and transferred to a polyvinylidene fluoride (PVDF) membrane. The samples were then blocked with 5% bovine serum albumin (BSA) for 15 min followed by incubating with according primary antibodies at 4 ${}^{\circ }C$ overnight. Subsequently, the samples were rinsed using PBS three times and incubated with respective secondary antibodies at 4 ${}^{\circ }C$ for 2 h. The samples were then washed with PBS and observed using a luminescence/fluorescence imaging system, and the bands were recorded.

### 2.8. Animal Models

Six to ten-week old of C57BL/6 mice were obtained from BioLASCO Taiwan Co., Ltd., Taipei, Taiwan.

#### 2.8.1. The Subcutaneous Tumor Models

In total, 5 × 10^6^ GL261 glioma cells in 100 μL PBS were injected into the subcutaneous tissues of the left legs of the C57BL/6 mice. Successful inductions of 100 mm^3^ subcutaneous tumors were seen in 14 days. The tumor-bearing mice were randomly divided into different treatment groups. On day 14, the first treatment included intraperitoneal injection of 2 μg mL^−1^ OMV and/or 200 μg mL^−1^ Au–OMV (10 μL in total) with or without 2 Gy of radiotherapy. The treatments were given every 3 days for 5 times. The tumor sizes were measured before and after each treatment every 4 days.

#### 2.8.2. The Brain Tumor Models (In Situ)

In total, 5 × 10^6^ GL261 glioma cells were injected into 25 brains of C57BL/6 mice through a specific locator. On day 5, after confirming successful induction of brain tumors using a non-invasive in vivo imaging system (IVIS) or MRI, mice were randomly divided and treated with 2 μg mL^−1^ OMV and/or 200 μg mL^−1^ Au–OMV (10 μL in total) using in situ injection. On the other day, 2 Gy of radiotherapy was given with an interval of 2–3 days, 5 times. Tumor sizes were evaluated using IVIS and MRI on days 12, 15, and 18.

## 3. Results

### 3.1. Au–OMV Complex

**Figure 1:**
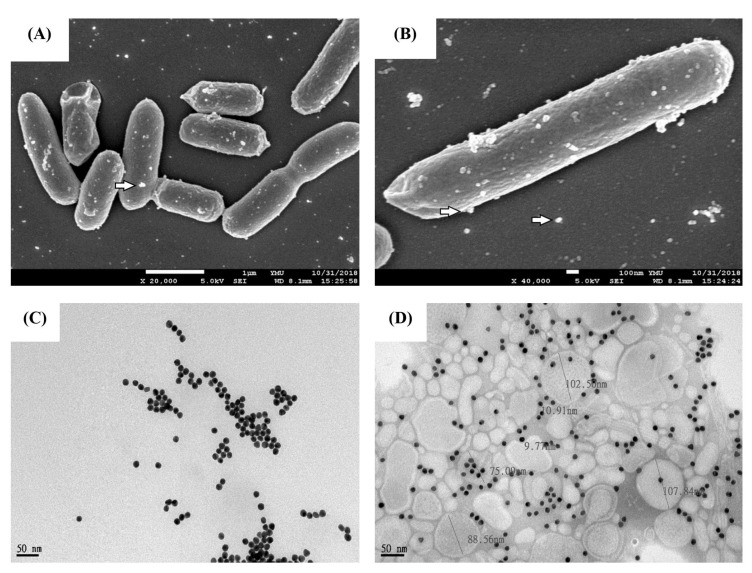
Characterizations of Au–OMV nanoparticles. (**A**,**B**) Scanning electron microscope image (SEM) of OMV production by *E. coli*. (**C**,**D**) TEM images of Au and Au–OMV.

The average diameter of synthetic Au-nanoparticles was 18 nm, and the sizes of these particles were homogeneous with a polydispersity index (PDI) equal to 0.073 (Table 1). The average diameter of OMVs was 126 nm. During the preparation of Au–OMV, solutions containing Au and OMV particles were mixed thoroughly in a homogenizer. As both Au and OMV particles were negatively charged, they could be mixed well to form a stable co-suspension solution without forming aggregations of particles (Figure 1).

### 3.2. Selective Cytotoxicity of OMV to Glioma Cells at Concentration ≥ 2 μg mL^−1^

**Figure 2:**
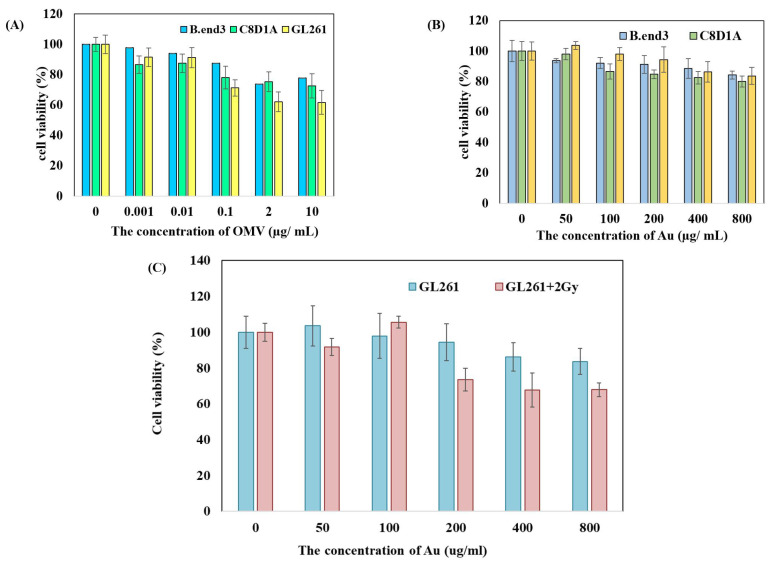
Toxicity of (**A**) OMV and (**B**) Au to B.end3 mouse brain endothelial cells, C8D1A mouse astrocytes, and GL261 mouse glioma cells. (**C**) Au augmented the radiotoxic effect towards GL261 mouse glioma cells.

OMVs with concentrations ranging from 0 to 10 μg mL^−1^ were incubated with B.end3 mouse brain endothelial cells, C8D1A mouse astrocytes, and GL261 mouse glioma cells at 37 ${}^{\circ }C$ for 24 h. When the cells were cultured with OMV concentrations greater than or equal to 2 μg mL^−1^, the survival rate of GL261 glioma cells reduced to 60%, whereas the survival rates of B.end3 endothelial cells and C8D1A astrocytes remained higher than 80% (Figure 2A).

### 3.3. AuNPs Were Nontoxic to Cells Unless Exposed to Radiotherapy at a Concentration of 200 μg mL^−1^

AuNPs with concentrations ranging from 0 to 800 μg mL^−1^ were incubated with B.end3 mouse brain endothelial cells, C8D1A mouse astrocytes, and GL261 mouse glioma cells at 37 ${}^{\circ }C$ for 24 h. The survival rates of all three types of cells were above 80% (Figure 2B). If AuNps-treated GL261 cells were exposed to 2 Gy of radiotherapy, the survival rate of cells was reduced by 30% when the AuNPs’ concentration reached 200 μg mL^−1^. However, with the concentration increased up to 800 μg mL^−1^, the cytotoxic effect remained at 30%, which showed the limitation of the augmented radiotherapy effect of AuNPs (Figure 2C). The cytotoxicity analysis showed that AuNPs were nontoxic to cells (Figure 2B) unless exposed to radiotherapy at a concentration of 200 g mL${}^{-1}$ (Figure 2C; i.e., showing selective toxicity to the GL261 cells). Therefore, a dose of 2 μg mL^−1^ OMV and/or 200 μg mL^−1^ Au–OMV was chosen for the following animal studies.

### 3.4. GL261 Cell Viability Reduced Significantly by Combination of Radiotherapy and Au–OMV Treatment When Co-Cultured with Macrophages

**Figure 3:**
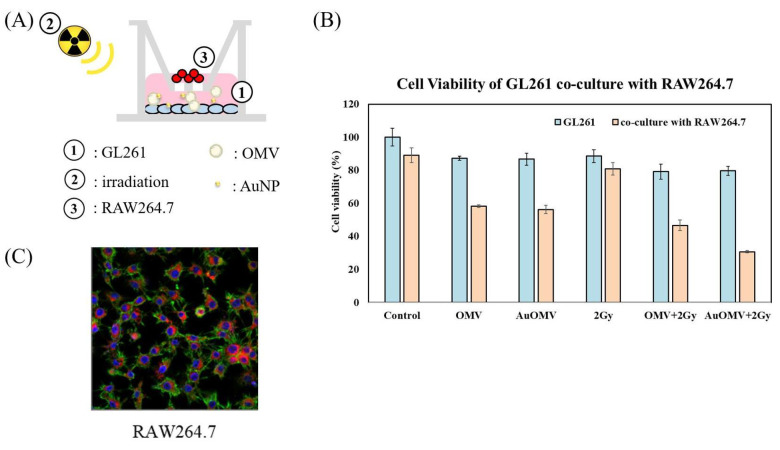
(**A**) GL261 glioma cells (in lower well) were treated with various treatments and co-cultured with RAW 264.7 Macrophages (in upper well) using 0.4 μm pored transwells. (**B**) GL261 cell viability in response to various treatments when co-cultured for 24 h with RAW 264.7 cells. (**C**) Co-culture in transwell system with a 3 μm porous membrane at the bottom of the upper chamber. Migrations of RAW 264.7 mouse macrophages towards glioma cells (chemoatxis of microphages) were noted and were labeled with VybrantTM DiD (red) in the lower chamber.

Cultured GL261 glioma cells were treated with various treatments and co-cultured with RAW 264.7 macrophages using 0.4 μm pored transwells which allowed only cytokines to move, not macrophages (Figure 3A). GL261 cell viability did not show significant change if they were not co-cultured with macrophages. However, the survival rate of glioma cells reduced to 60% when the co-culture systems were treated with OMV only, and further reduced to 30% if the treatments combined both OMV and AuNPs with 2 Gy of radiotherapy (Figure 3B).

### 3.5. Chemotaxis of Macrophages to Glioma Cells Was Induced by Au–OMV in Combination with Radiotherapy

**Figure 4:**
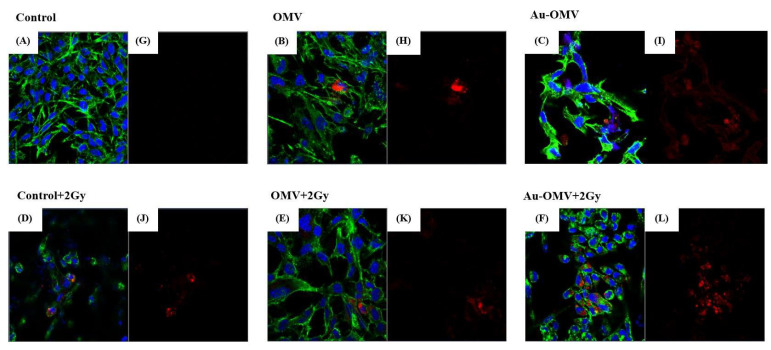
Confocal microscopy of immunofluorescence imaging of the GL 261 glioma cells co-cultured with RAW 264.7 macrophages. (**A**,**G**) Control and treatments with (**B**,**H**) OMV, (**C**,**I**) Au–OMV, (**D**,**J**) 2 Gy X-ray, (**E**,**K**) OMV + 2 Gy X-ray, and (**F**,**L**) Au–OMV + 2 Gy X-ray. ( immunofluorescences: H33342/DAPI (blue for nucleus), phalloidin (green for actin), and VybrantTM DiD (red for macrophages).

GL261 glioma cells were treated with various treatments and co-cultured with RAW 264.7 macrophages for 24 h using 3 μm transwells, which allowed the migration of macrophages. The migrations of macrophages towards glioma cells (chemotaxis of macrophages) were observed under confocal microscopy using VybrantTM DiD-stained (red) macrophages (Figure 3C). The chemotaxis of macrophages largely increased when glioma cells were treated with Au–OMV combined with 2-Gy radiotherapy (Figure 4). The degrees of chemotaxis of macrophages in response to various treatments seemed correlated with the changes of glioma cell viability to the same treatment.

### 3.6. ROS Production of Glioma Cells Greatly Increased with Au–OMV in Combination with Radiotherapy

**Figure 5:**
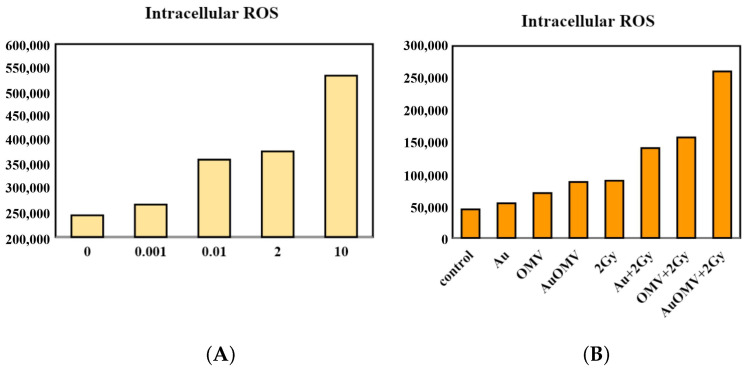
Intracellular ROS production of G261 glioma cells in response to (**A**) different concentrations of OMV and (**B**) various treatments.

It is well known that metal-based nanoparticles’ induction of cancer cell death is related to the generation of ROS [12]. However, ROS production of cancer cells related to OMV has not been explored. In this study, ROS production in GL261 glioma cells was induced by OMV administration, and the reaction was dose-dependent (Figure 5A). With the combination of Au–OMV and radiotherapy, ROS production increased as much as five times the control (Figure 5B).

**Figure 6:**
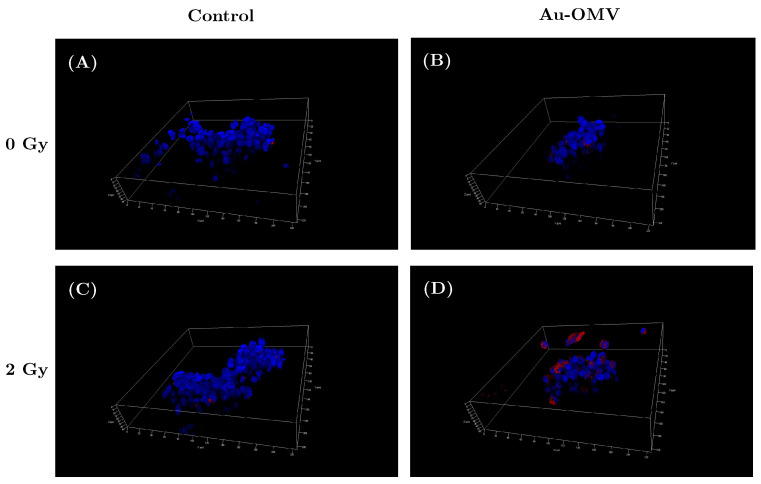
ROS production of live G261 glioma cells grown on 3D spheres in control (**A**), in response to (**B**) Au–OMV, (**C**) 2-Gy radiotherapy, and (**D**) the combination of Au–OMV and radiotherapy under confocal microscopy. The live cells were labeled with H33342/DAPI (blue for nucleus), phalloidin (green for actin), and CellROXTM Deep Red Reagent for oxidative stress detection (pink for ROS).

To simulate 3D tumor behavior, we built a 3D sphere grown with GL261 glioma cells. Briefly, GL261 glioma cells were grown in Nunclon Sphera microplates at 10,000 cells/well, and the live cells were labeled with H33342/ DAPI (blue for nucleus), phalloidin (green for actin), and CellROX Deep Red Reagent for oxidative stress detection (pink). Under confocal microscopy (Figure 6), a mild increase in ROS production was observed when the glioma cells were treated with Au–OMV (Figure 6B) and 2-Gy radiotherapy (Figure 6C). The greatest increase of ROS production was detected while 3D spherical glioma cells were treated with Au–OMV and 2-Gy radiotherapy (Figure 6D). The results in 3D spheres were correlated with those in the culture discs.

### 3.7. TNF-α Expression in the GL261 and RAW 264.7 Co-Culture Was Increased by Combining Radiotherapy with Au–OMV

**Figure 7:**
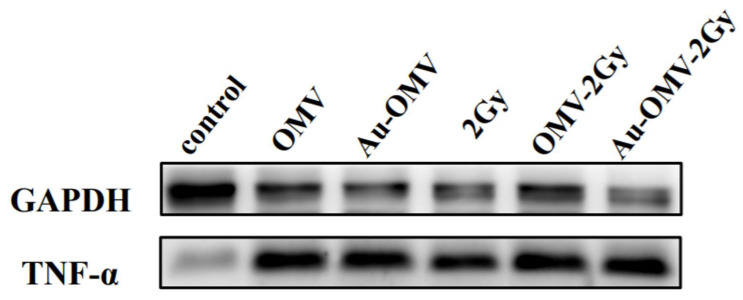
Western blot of TNF-α released from RAW 264.7 macrophages co-cultured with GL261 glioma cells. GAPDH was used as a loading control.

GL261 glioma cells were treated with various treatments and co-cultured with RAW 264.7 macrophages for 24 h using 0.4 μm transwells, which allowed only cytokines and not macrophages to move. TNF-α protein expression in cell lysates was evaluated by Western blots. Increased production of TNF-α protein after various treatments was found in the lysate of cells treated with Au–OMV combined with radiotherapy (Figure 7).

### 3.8. In Vivo Glioblastoma Animal Models Successfully Treated with Intraperitoneal Injection and In Situ Injection of Au–OMV Combined with 2 Gy Radiotherapy

#### 3.8.1. The Subcutaneous Tumor Models

**Figure 8:**
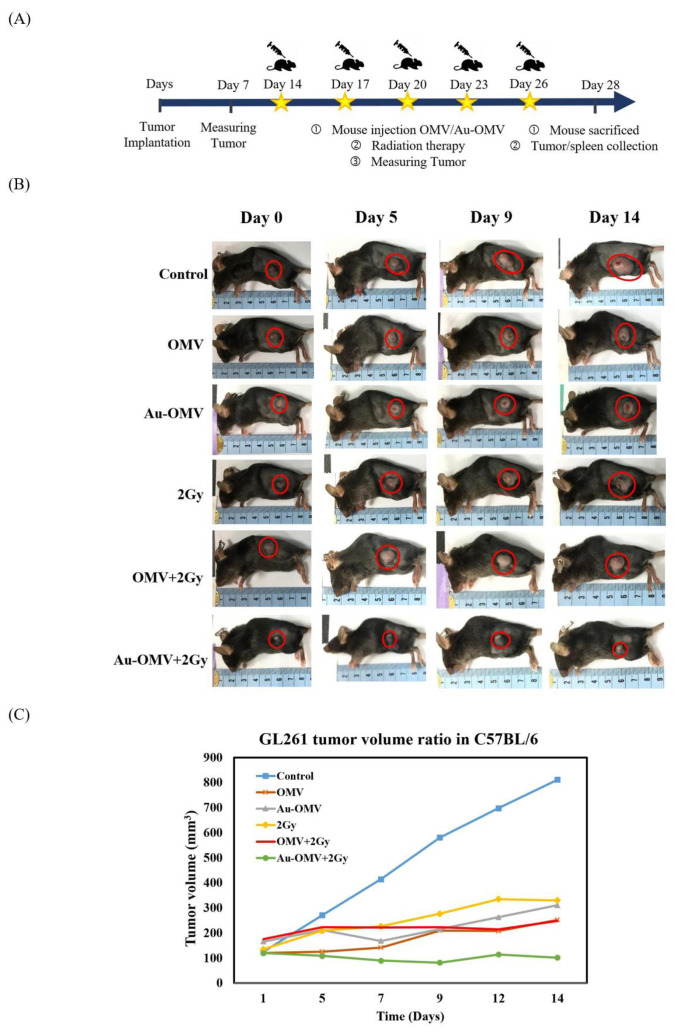
(**A**) Schematic diagram of subcutaneous OMV or Au–OMV injections with radiation therapy for GL261 glioma tumor-bearing mice. (**B**,**C**) Tumor volume kept growing in the control group, but the growth was suppressed in all treatment groups, especially in the group treated with Au–OMV + 2 Gy radiotherapy, whose tumors did not grow, even at the end of the experiment.

In this step, 5 × 10^6^ GL261 glioma cells were injected into the subcutaneous tissues of the left legs of the C57BL/6 mice. The successful induction of 100 mm^3^ subcutaneous tumors was seen in 14 days. The tumor-bearing mice were randomly divided into different treatment groups. Treatment groups were given intraperitoneal injections of 2 μg OMV or 200 μg Au–OMV with or without 2-Gy radiotherapy every 3 days, five times (Figure 8A). The size of each tumor was measured before and after treatment (Figure 8B). The tumor volume steadily increased with time in the control group, but was successfully suppressed by treatments, especially in the group treated with Au–OMV with 2-Gy radiotherapy (Figure 8C). Growth of tumors was suppressed in all treatment groups, especially in the group treated with Au–OMV + 2 Gy radiotherapy, whose tumors did not grow even at the end of the experiment.

#### 3.8.2. The In Situ Brain Tumor Models

**Figure 9:**
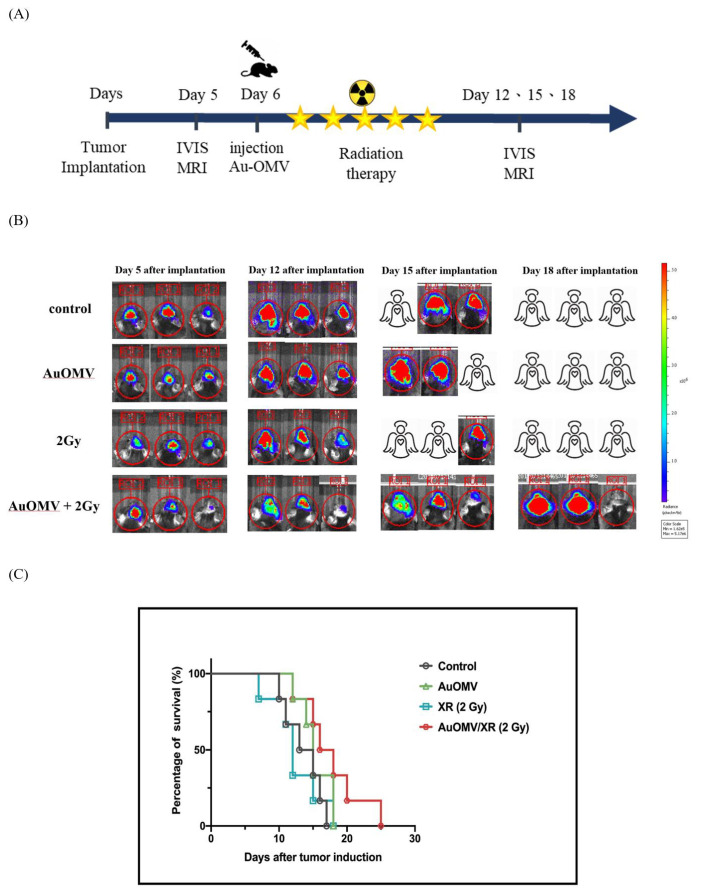
(**A**) Schematic diagram of intracranial Au–OMV injection with radiation therapy for GL261 glioma tumor-bearing mice. (**B**) Assessment of the IVIS imaging system as a method for monitoring tumor growth in a GL261 in situ tumor mode. IVIS imaging of mouse brain tumors on days 5, 12, 15, and 18 after implantation of tumor cells; imaging results of three mice in each group. All images were at the same scale. (**C**) Kaplan–Meier survival curves of control and treatment groups (N > 5 per arm).

In addition, 5 × 10^6^ GL261 glioma cells were injected into the brains of C57BL/6 mice through a specific locator. On day 5, after confirming successful induction of brain tumors using a noninvasive in vivo imaging system (IVIS), mice were randomly divided into groups and treated with Au–OMV in a 10 μL solution using in situ injection on day 7. To some groups, 2-Gy radiotherapy was given. Tumor sizes were evaluated using IVIS on days 12, 15, and 18 (Figure 9A). Only when mice were treated with Au–OMV combined with 2-Gy radiotherapy was tumor growth suppressed at the beginning of the treatment. If tumor size was small, eradication of the tumor was noted after complete treatment. Only when tumor-bearing mice were treated with Au–OMV combined with 2-Gy radiotherapy could they live more than 18 days after tumor implantation (Figure 9B). The survival rate after 18 days was 35% for the Au–OMV with 2-Gy radiotherapy group (Figure 9C).

## 4. Discussion

Glioblastoma is known as the most difficult brain tumor to remove because of its infiltrating nature. In addition, the difficulty of reaching the tumor with chemotherapeutic drugs due to the blood brain barrier (BBB) and the limitations of radiotherapy in eradicating radio-resistant glioblastoma cells further prevent the development of effective treatment. To overcome these hurdles, the use of nano-particulate anti-GBM drugs has been suggested [13]. Before our study, Au-related nanoparticles had been used to radio-sensitize glioma stem cells [14], and OMVs had been evaluated for antitumor treatments in multiple tumors, but never been studied for the treatment of glioblastoma.

In this study, we successfully synthesized a complex of two specific nanoparticles, AuNPs and OMVs, to form Au–OMVs (Figure 1). Au–OMVs improve the GBM treatment via several different mechanisms. The efficacy of radiotherapy against GBM tumor is increased due to its radio-sensitizing effect, and the immune mechanisms relying on activation of anti-tumor cytokines and immune cells and local production of ROS are enhanced. At first, we evaluated our synthesized AuNPs and OMVs individually. OMVs had specific cytotoxicity to mouse glioma cells and were relatively safe to endothelial cells and astrocytes. At concentrations above 2 μg mL^−1^, GL261 cells’ survival rate reduced to 60% as compared to 80% of B.end3 and C8D1A cells (Figure 2A). Up to 800 μg mL^−1^ of AuNPs, glioma cells, and normal endothelial cells and astrocytes had survival rates of at least 80% (Figure 2B). AuNPs had a radio-sensitizing effect at the concentration of 200 g dL^−1^, causing a 30% reduction of GL261 cell survival, and the augmentation was not dose-related (Figure 2C). In general, both OMV and Au particles were relatively safe to normal endothelial cells and astrocytes and had mild cytotoxic effects on glioma cells at specific concentrations.

The histopathology of glioblastoma is characterized by significant infiltration of resident microglia and peripheral macrophages in the tumor and pervasive infiltration of tumor cells into the healthy surroundings of the tumor. Microglia and macrophages are the main innate immune cells of the central nervous system. These macrophage populations infiltrate the brain tumor area and constitute up to 50% of non-neoplastic cells, presenting an opening for therapeutic strategies [15]. It has been speculated that the recruitment of tumor-associated microglia and macrophages by tumor cells could be a potential approach for drug delivery [16,17]. Before our study, it was clear that macrophages play a significant role in the pathophysiology of glioblastomas. In our study, we co-cultured RAW 264.7 macrophages and G261 glioma cells using transwells with different pore sizes to simulate the microenvironment of the glioblastoma and evaluate the immunological responses to different treatments, especially our new Au–OMVs. Using 0.4 μm transwells, macrophages cultured in the transwell-insert influenced glioma cell growth without migration to the lower compartment (Figure 3A). The survival of GL261 glioma cells reduced to 30% when the co-cultured systems were treated with Au–OMV combined with 2-Gy radiotherapy (Figure 3B).

Reactive oxygen species (ROS) produced in eukaryotic cells through aerobic metabolism have evolved as regulators of important signaling pathways. ROS are generated after exposure to physical agents and after chemotherapy and radiotherapy [18]. It was not surprising that intracellular ROS increased after radiotherapy. However, in spite of limited literature, there was still evidence showing that OMVs dramatically increased the production of MCP-1 (macrophage/monocyte chemoattractant protein-1) and MCP-2 (macrophage/monocyte chemoattractant protein-2) cytokines from neurons [19]. The elevated secretion of MCP-1 was then associated with changes in oxidative stress [20,21]. In our study, intracellular ROS production also increased with OMV treatment alone in a dose-dependent way (Figure 5A). OMVs are known to have immunomodulatory function [22]. The ROS production of GL261 glioma cells in response to OMV treatment might have been related to the immunomodulation. To our knowledge, this is the first study to demonstrate OMVs’ effect on ROS production in glioma cells. Furthermore, treatment with Au–OMV caused more ROS production than OMV alone. Both Au and OMVs increased the intracellular ROS production in response to 2-Gy radiotherapy. The complex of Au–OMV in combination with 2-Gy radiotherapy induced the greatest intracellular ROS production (Figure 5B). The ROS production was mostly induced by the combination of Au–OMV with 2 Gy radiotherapy, not only in 2D culture, but also in 3D culture (Figure 6). ROS have been implicated as mediators of cell survival and cell death triggered by TNF-α signaling [23,24].

TNF-α was thought to be produced primarily by macrophages [25]. Using 3 μm transwells co-culture system, we labeled macrophages with red fluorescence (Figure 3C) and observed macrophage migration (chemotaxis) under a confocal microscope. The complex of Au–OMV in combination with 2-Gy radiotherapy made the most macrophages migrate from the upper compartment to the lower compartment (Figure 4). The reduction of GL261 glioma cell survival seemed correlated with macrophage chemotaxis to glioma cells. Not only did macrophage chemotaxis increase with Au–OMV and 2-Gy radiotherapy treatment, but macrophage-related cytokine also increased. Using a 0.4 μm transwells co-culture system and Western blotting, we also found that the protein levels of TNF-α increased after the system was treated with Au–OMV with 2-Gy radiotherapy (Figure 7).

Based on the successful in vitro results, we tested the Au–OMV complex on two in vivo animal models with C57BL/6 mice. First, for easier tumor observation and more convenient drug administration, we created the subcutaneous tumor mass using GL261 glioma cells were implanted on the left thighs of C57BL/6 mice. Treatment groups were given intraperitoneal injections of 2 μg OMV or 200 μg Au–OMV with or without 2-Gy radiotherapy every 3 days, five times (Figure 8A). The tumor volume steadily increased with time in the control group, but was successfully suppressed by treatments, especially in the group treated with Au–OMV and 2-Gy radiotherapy. After 2 weeks, tumor volume barely changed in tumor-bearing mice treated with Au–OMV and 2-Gy radiotherapy in comparison to an eight-fold increase in tumor volume of the control mice (Figure 8C).

Encouraged by the successful subcutaneous model results, we created an in situ brain tumor animal model by implanting GL261 cells into the brain using a specific locator. The in situ brain tumor model was more challenging for tumor size measurements and drug administration. For tumor measurements, we injected D-luciferin preparation into peritoneum and observed tumor size by IVIS. Successful tumor reduction was observed in the in situ tumor-bearing mice treated with Au–OMV and 2-Gy radiotherapy; control mice or mice treated with only Au–OMV or 2-Gy radiotherapy experienced tumor growth or death. If tumor size was small, eradication of the tumor was noted at the end of the experiment. However, tumor regrowth was noted in some mice treated with Au–OMV and 2-Gy radiotherapy, whereas non-treated mice or mice that received a single treatment died (Figure 9B). Non-treated, in situ, GL261 tumor-bearing mice had very short lives (ranged from 15–18 days). Although tumor regrowth was noted, tumor-bearing mice treated with Au–OMV and 2-Gy radiotherapy had longer survival (Figure 9C). More experimentation is needed to evaluate whether higher dosages or repeated treatments will help to prolong the disease-free time.

## 5. Conclusions

In this study, the combination of Au–OMV and radiotherapy had a specific cytotoxic effect on GL261 glioma cells which may have been related to intracellular ROS production, chemotaxis of macrophages, and TNF-α production. We also created two animal models using GL261 cells implanted in the subcutaneous tissues or brains of C57BL/6 mice. As a result, suppression of tumor growth was noted in all tumor-bearing mice treated with both Au–OMV and radiotherapy. Au–OMV opened up a new avenue for the treatment of glioblastoma using low-dose combination radiotherapy. However, further studies on the detailed mechanisms of OMVs are important for more understanding. Such understanding could be crucial for the future development of chemically conjugated Au–OMV preparations. As Au–OMV-based therapy uses different mechanisms from temozolomide and bevacizumab in the treatment of glioblastoma, more studies are necessary to compare these therapeutic agents and investigate the feasibility of combinations of them in chemoradiation strategies of glioblastoma management.

## Figures and Tables

**Table 1:** The size distributions of Au and OMV particles.

Nanoparticles	Size (nm)	PDI
Au	17.85	0.073
OMV	126	0.238
Au–OMV	42	0.521
